# Supplementary material for: Plasmodium knowlesi clinical isolates from Malaysia show extensive diversity and strong differential selection pressure at the merozoite surface protein 7D (MSP7D)
Source: Malar J. 2019 Apr 29;18:150. doi: 10.1186/s12936-019-2782-2 (PMC6489361; doi:10.1186/s12936-019-2782-2)
Supplement: Supplementary file 1 — Additional file 1: Table S1. Study samples and origin. [file 12936_2019_2782_MOESM1_ESM.docx]

**Additional file 1 Table S1. Study samples and origin**

| **No.** | **Sample** | **Area** | **Year** |
| --- | --- | --- | --- |
| 1 | ERR274221 | MB, Sarikei | 2012/3 |
| 2 | ERR274222 | MB, Sarikei | 2012/3 |
| 3 | ERR366426 | MB, Sarikei | 2012/3 |
| 4 | ERR985374 | MB, Betong | 2012/3 |
| 5 | ERR985376 | MB, Betong | 2012/3 |
| 6 | ERR985377 | MB, Betong | 2012/3 |
| 7 | ERR985378 | MB, Betong | 2012/3 |
| 8 | ERR985379 | MB, Betong | 2012/3 |
| 9 | ERR985380 | MB, Betong | 2012/3 |
| 10 | ERR985381 | MB, Betong | 2012/3 |
| 11 | ERR985382 | MB, Betong | 2012/3 |
| 12 | ERR985383 | MB, Betong | 2012/3 |
| 13 | ERR985384 | MB, Betong | 2012/3 |
| 14 | ERR985385 | MB, Kapit | 2012/3 |
| 15 | ERR985386 | MB, Kapit | 2012/3 |
| 16 | ERR985387 | MB, Kapit | 2012/3 |
| 17 | ERR985388 | MB, Kapit | 2012/3 |
| 18 | ERR985390 | MB, Kapit | 2012/3 |
| 19 | ERR985392 | MB, Kapit | 2012/3 |
| 20 | ERR985393 | MB, Kapit | 2012/3 |
| 21 | ERR985394 | MB, Kapit | 2012/3 |
| 22 | ERR985396 | MB, Kapit | 2012/3 |
| 23 | ERR985397 | MB, Kapit | 2012/3 |
| 24 | ERR985404 | MB, Kapit | 2012/3 |
| 25 | ERR985406 | MB, Kapit | 2012/3 |
| 26 | ERR985408 | MB, Kapit | 2012/3 |
| 27 | ERR985411 | MB, Betong | 2012/3 |
| 28 | ERR985416 | MB, Kapit | 2012/3 |
| 29 | ERR985417 | MB, Kapit | 2012/3 |
| 30 | ERR985418 | MB, Kapit | 2012/3 |
| 31 | MR4 | P. Malaysia |  |
| 32 | Malayan Strain Pk1A ( PKNOH_S09532900) | P. Malaysia |  |
| 33 | H-strain(PKNH_1266000) | P. Malaysia |  |
| 34 | Hackeri Strain (KU307327) |  |  |
| 35 | ERR985389 | MB, Kapit | 2012/3 |
| 36 | Phillipine (SRR2225573) |  |  |

P Peninsular; MB Malaysian Borneo

P: Peninsular
