# Supplementary figures and images for: Plasmodium knowlesi clinical isolates from Malaysia show extensive diversity and strong differential selection pressure at the merozoite surface protein 7D (MSP7D)
Source: Malar J. 2019 Apr 29;18:150. doi: 10.1186/s12936-019-2782-2 (PMC6489361; doi:10.1186/s12936-019-2782-2)

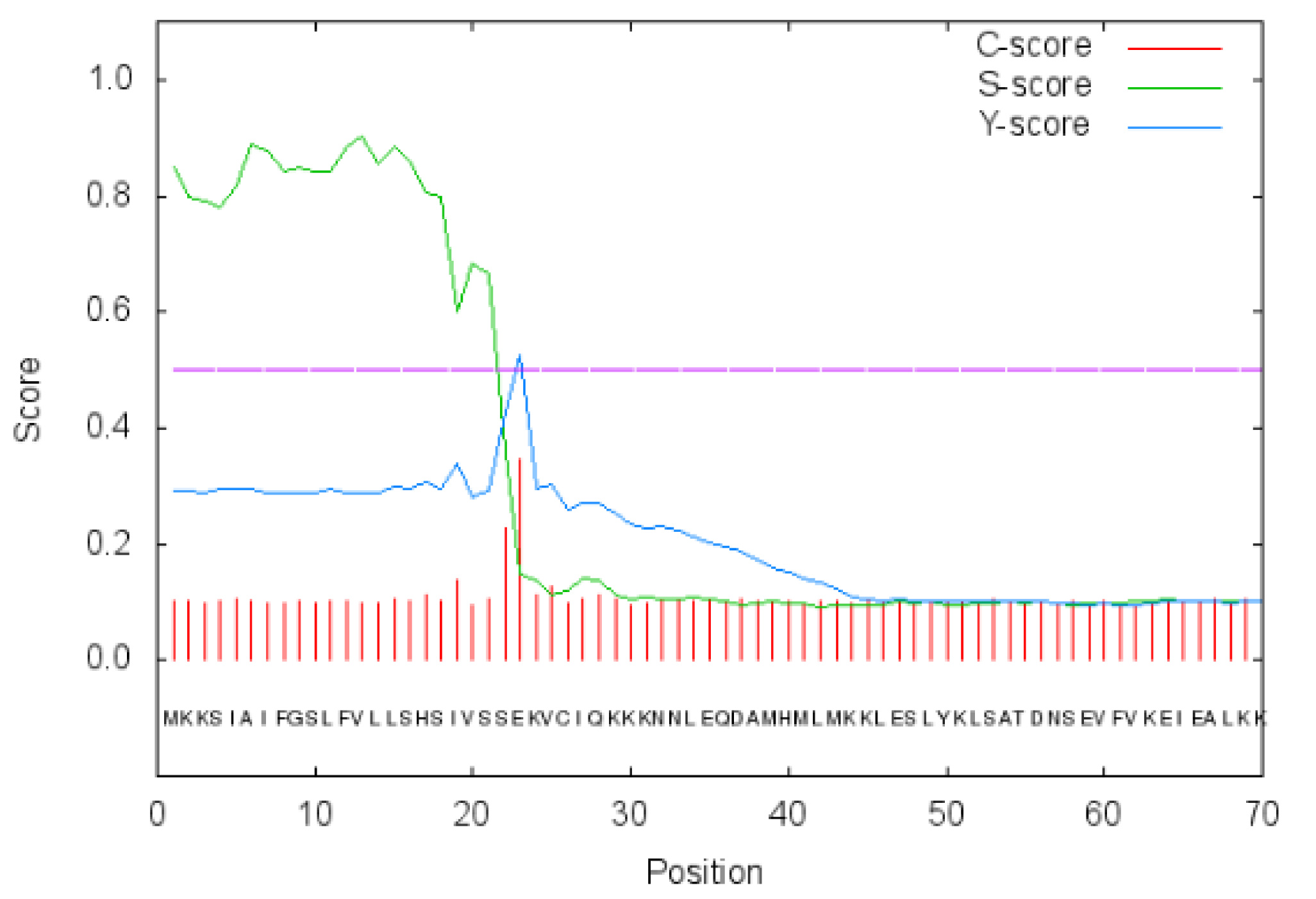

Supplement: Supplementary file 2 — Additional file 2. Signal peptide identifed by Signal IP server. [file 12936_2019_2782_MOESM2_ESM.png]
